# Role of the Hydroxyl Radical-Generating System in the Estimation of the Antioxidant Activity of Plant Extracts by Electron Paramagnetic Resonance (EPR)

**DOI:** 10.3390/molecules27144560

**Published:** 2022-07-17

**Authors:** Daniele Sanna, Angela Fadda

**Affiliations:** 1 Istituto di Chimica Biomolecolare, Consiglio Nazionale delle Ricerche, Traversa la Crucca 3, I-07100 Sassari, Italy; 2 Istituto di Scienze delle Produzioni Alimentari, Consiglio Nazionale delle Ricerche, Traversa la Crucca 3, I-07100 Sassari, Italy

**Keywords:** Fenton reaction, hydroxyl radical, EPR, DMPO, plant extracts

## Abstract

The scavenging activity of hydroxyl radicals, produced by the Fenton reaction, is commonly used to quantify the antioxidant capacity of plant extracts. In this study, three Fenton systems (Fe/phosphate buffer, Fe/quinolinic acid and Fe/phosphate buffer/quinolinic acid) and the thermal degradation of peroxydisulfate were used to produce hydroxyl radicals; the hydroxyl radical scavenging activity of plant extracts (ginger, blueberry juices and green tea infusion) and chemical compounds (EGCG and GA) was estimated by spin trapping with DMPO (5,5-dimethyl-1-pyrroline N-oxide) and EPR (Electron Paramagnetic Resonance) spectroscopy. Phosphate buffer was used to mimic the physiological pH of cellular systems, while quinolinic acid (pyridine-2,3-dicarboxylic acid) facilitates the experimental procedure by hindering the spontaneous oxidation of Fe(II). The EC_50_ (the concentration of chemical compounds or plant extracts which halves the intensity of the DMPO–OH adduct) values were determined in all the systems. The results show that, for both the chemical compounds and the plant extracts, there is not a well-defined order for the EC_50_ values determined in the four hydroxyl radical generating systems. The interactions of phosphate buffer and quinolinic acid with the antioxidants and with potential iron-coordinating ligands present in the plant extracts can justify the observed differences.

## 1. Introduction

Among ROSs (Reactive Oxygen Species), the hydroxyl radical ^•^OH is one of the most reactive and dangerous, being able to damage cellular systems such as DNA, proteins and lipids [1]. Based on the literature data [2,3], plant extracts are capable of reacting with the hydroxyl radical, diminishing its damaging effects. 

Therefore, the measurement of the hydroxyl radical scavenging activity of vegetable matrices is important from a biological point of view for the selection of effective extracts suitable for nutraceutical or pharmacological applications. 

To measure the hydroxyl radical scavenging activity, this radical, being an extremely reactive species, should be prepared in situ, and one of the approaches takes advantage of the Fenton reaction. The reaction involves Fe(II) and hydrogen peroxide, in agreement with Equation (1)

Fe(II) + H_2_O_2_ → Fe(III) + OH^−^ + ^•^OH (1)

To mimic the physiological pH, a buffer is usually used. This reaction seems very simple but is extremely sensitive to the experimental conditions under which it is performed. In fact: (i) Fe(II) is readily oxidized to Fe(III) by atmospheric oxygen, and the reaction becomes faster with increasing pH values; (ii) the redox potential of the couple Fe(II)/Fe(III) is influenced by the ligands (and, among these, also by buffers such as phosphate) present in the vegetable matrices, and the oxidation to Fe(III) is favored by the ligands stabilizing this oxidation state; (iii) the hydrogen peroxide, being an oxidant, can react directly with the antioxidants with a mechanism not involving Fe(II).

The hydroxyl radical, being an extremely reactive species, cannot be directly detected by EPR. To be detectable, it could be entrapped by DMPO to form a relatively stable paramagnetic adduct, DMPO–OH. DMPO is a redox inactive nitrone spin trap that allows for the identification of the trapped radicals since the hyperfine coupling constants (*a*_N_, *a*_H_) of its adducts strongly depend on the structure of the trapped radical [4]. A large amount of data exist for DMPO adducts, and only two alternate reactions leading to radical adduct artifacts are known. The first, inverted spin trapping, assumes the oxidation of DMPO to its radical cation followed by nucleophilic addition to form a paramagnetic adduct. It is, however, of limited importance because of the very high oxidation potential of DMPO (1.63 V) [4]. This mechanism can be observed during the thermal degradation of peroxydisulfate. The sulfate anion radical is capable of oxidizing DMPO, but after the addition of the nucleophilic water, it generates the same DMPO–OH adduct formed after the trapping of the hydroxyl radical [5]. The second mechanism of potential artifact generation concerns the Forrester–Hepburn reaction, which assumes the addition of a nucleophilic to DMPO followed by its oxidation [4]. In the presence of Lewis acids such as Fe(III), water molecules can be added to DMPO, which is then oxidized to form the DMPO–OH paramagnetic adduct. However, when the reaction is carried out in buffers or in the presence of iron chelators, this reaction is effectively suppressed [6]. Based on these considerations and the conditions applied in this paper, the DMPO can be considered a reliable spin trap for plant extract hydroxyl radical activity determinations.

We previously examined the influence of pH, buffers and DMPO concentration on the production and entrapment of the hydroxyl radicals obtained with a Fenton reaction [7]. To facilitate the experimental procedure and hinder the spontaneous oxidation of Fe(II) solutions exposed to air, the use of pyridine-2,3-dicarboxylic acid (quinolinic acid, Quin) was proposed [7,8,9]. The hydroxyl radical scavenging activity of the same amount of green tea, orange juice and asparagus extracts was examined in three different Fenton systems, where the Fe(II)/phosphate buffer, Fe(II)/Quin and Fe(II)/Quin/phosphate buffer were used. It was observed that the percentage of the inhibition of the extracts was dependent on the Fenton system, highlighting the influence of the extract composition on the generation and trapping of the hydroxyl radical. 

Another approach to measuring the hydroxyl radical scavenging activity is based on peroxydisulfate, which is stable at room temperature but decomposes at 60 °C, generating hydroxyl radicals [5], according to Equation (2):

K_2_S_2_O_8_—(60 °C)→ 2 K^+^ + 2 SO_4_^−^^•^ SO_4_^−^^•^ + H_2_O → HSO_4_^−^ + ^•^OH (2)

In this system, there is no iron, and, therefore, there cannot be any interference by the potential coordinating ligands present in the extracts. On the contrary, peroxydisulfate, being an oxidant, can react directly with the antioxidants without the involvement of the hydroxyl radical.

In this paper, we estimated the hydroxyl radical scavenging activity of blueberry, ginger juices and green tea infusion. Two chemical compounds (epigallocatechin gallate, EGCG and gallic acid, GA) were also used for a comparison with plant extracts. The results were expressed as EC_50_ values, which are the concentration values that give 50% of the inhibition of the intensity of the DMPO–OH adduct. Four hydroxyl radical-generating systems—three based on the Fenton reaction (Fe(II)/phosphate buffer, Fe(II)/Quin and Fe(II)/Quin/phosphate buffer) and one based on the thermic decomposition of peroxydisulfate—were compared to highlight the interactions among the chemical composition of the extracts and the hydroxyl radical-generating systems and the effect of the composition on the final radical scavenging activity results (EC_50_ values). The purpose of this manuscript is to establish if the EC_50_ values of the hydroxyl radical scavenging activity for a specific matrix (juice, infusion or chemical compound) depend on the employed hydroxyl radical-generating system and, by comparison with single chemical compounds, if they depend on the matrix complexity or if they can be affected by the presence of iron-coordinating ligands present in the plant extract. In particular, our purpose is to verify whether, for each extract or single compound, the EC_50_ values obtained with the four hydroxyl radical-generating systems changed with the same order. This would mean that the composition of the extracts does not affect the hydroxyl radical-generating system.

## 2. Materials and Methods

### 2.1. Reagents and Solvents

K_2_S_2_O_8_ was purchased from Fluka (code 60489). Epigallocatechin gallate (EGCG: code E4268), Gallic acid (GA: code G7384) and FeSO_4_·7H_2_O (code 215422) were purchased from Sigma Aldrich. DMPO (5,5-dimethyl-1-pyrroline N-oxide) was purchased from Enzo Life and used without further purification. Water was purified with a Milli-Q system from Millipore (Millipore Corporation, Billerica, MA, USA) and deaerated before use.

### 2.2. Preparation of the Extracts

Fresh blueberries (*Vaccinium corymbosum* L. cv Brigitta Blue) were purchased at the local market. Blueberry juice was prepared by pressing the fresh blueberries. The fruit puree was centrifuged at 6000 rpm for 15 min, and the juice was further filtered with 0.45 µm cellulose filters. Juice samples were stored at −20 °C until analysis.

Ginger (*Zingiber officinale* L.) roots were purchased at the local market. Ginger juice was prepared by grinding, pressing and centrifuging (6000 rpm for 15 min) the ginger roots, which were previously peeled.

Green tea was obtained by the infusion of commercially available tea bags (Twinings—R. Twining and Company Limited, London) (2 g) for 5 min in 100 mL of distilled water at 80 °C. The cooled infusion was filtered under vacuum with a Whatman 113 filter. The concentration of this solution was formally considered as 2 g/100 mL or 20 mg/mL.

### 2.3. Preparation of the Antioxidant Compound Solutions

Epigallocatechin gallate (EGCG) solutions were prepared by dissolving in MilliQ water the proper amount of EGCG to obtain a stock solution with a final concentration of 2 mM. Gallic acid (GA) solutions were prepared by dissolving in MilliQ water the proper amount of GA to obtain a stock solution with a final concentration of 10 mM.

### 2.4. Determination of the Hydroxyl Radical Scavenging Activity with the Spin Trapping Method

The hydroxyl radical scavenging activity was determined with the spin trapping method coupled with Electron Paramagnetic Resonance (EPR) Spectroscopy. Four hydroxyl radical-generating systems—three based on the Fenton reaction and one based on the thermic decomposition of peroxydisulfate—were compared. The hydroxyl radical-generating systems based on the Fenton reaction used Fe(II)-sulphate or the Fe(II)-Quin complex as Fe(II) sources, according to our previous work [7], and phosphate buffer 20 mM (pH 7.4) to mimic the physiological conditions. Fe(II) solution was prepared by dissolving the proper amount of FeSO_4_·7H_2_O in degassed MilliQ water and bubbling with argon gas to hinder the spontaneous oxidation to Fe(III). The Fe(II)–Quin complex was prepared by solubilizing in water FeSO_4_·7H_2_O and pyridine-2,3-dicarboxylic acid (quinolinic acid, Quin) to obtain a ligand-to-metal ratio of 5/1 and a Fe(II) concentration of 0.1 mM. When pyridine-2,3-dicarboxylic acid is present, the bubbling of argon is unnecessary because the spontaneous oxidation of Fe(II) is prevented. In the Fenton reaction, Fe(II) reacts with hydrogen peroxide to produce a hydroxyl radical and a hydroxyl ion. A hydrogen peroxide solution, 9.8 mM, was prepared from an H_2_O_2_ concentrated solution 30% (*w/w*) and kept in an ice bath to avoid decomposition. The hydroxyl radicals were trapped with the nitrone spin trap DMPO. Diluted solutions of juices, tea infusion and chemical compounds were prepared in degassed MilliQ water, and these were bubbled with argon to avoid their oxidation.

In a reaction volume of 1 mL, the solutions were added in the following order (the final concentrations are reported in brackets): buffer phosphate (20 mM when present), water, juice, tea infusion or GA and EGCG, DMPO (0.6 mM), H_2_O_2_ (0.979 mM), Fe(II) (0.01 mM) or Fe(II)-Quin (Fe(II) 0.01 mM and Quin 0.05 mM).

The peroxydisulfate assay was carried out by mixing 50 μL of a 5 mM solution of K_2_S_2_O_8_ with 167 mL of a 3 mM solution of DMPO, along with variable amounts of solutions of juices, infusion or chemical compounds, to reach a final volume of 1 mL. These were kept at 60 °C in a water bath for 10 min and then transferred to the AquaX cell already inside the EPR cavity. The EPR spectra were recorded at room temperature.

A Bruker EMX spectrometer operating at the X-band (9.4 GHz) and equipped with an HP 53150A microwave frequency counter was used to detect the DMPO-OH adduct signals by using a Bruker AquaX capillary cell. During the sample measurements, the Q (the quality factor of the resonator) value was kept constant, thus allowing for quantitative comparisons of the intensity of the EPR signals, in agreement with ref. [10]. The influence of other factors (filling factor, radio frequency power, etc.) was considered negligible because these were the same for all the measurements.

The results are expressed as EC_50_, which corresponds to the concentration of the extract or pure compound that halves the number of radicals produced by the hydroxyl radical-generating systems.

The EC_50_ values were calculated by plotting against juices/infusion/chemical compounds concentration the inhibition percentage values obtained as follows: percent of inhibition = 100 × (I_0_ − I_S_)/I_0_, where I_0_ is the intensity of the signal of the spin adduct without the juices/infusion/chemical compounds (i.e., the intensity of the blank) and I_S_ is the intensity of the signal of the adduct after the reaction with variable amounts of the juices/infusion/chemical compounds. The blanks were used as the references for the maximum intensity and were prepared as the samples, except they did not contain the vegetable matrices. Therefore, we used four different blanks for comparison; three of them contain Fe(II), H_2_O_2_ and DMPO and can contain quinolinic acid and/or phosphate buffer, while the blank used for the peroxydisulfate assay contains only K_2_S_2_O_8_ and DMPO.

### 2.5. EPR Spectrometer Settings

The EPR instrument was set under the following conditions: modulation frequency, 100 kHz; modulation amplitude, 1 G; receiver gain, 1 or 5.02 × 10^5^; sweep time, 168 s; microwave power, 20 mW. This microwave power, using the Bruker ER 4119HS resonator, is below the saturation level. The EPR spectra were recorded at room temperature immediately after the preparation of the reaction mixture. Only one scan was acquired for each sample, keeping all the experimental parameters constant.

The intensity of the spin adduct DMPO–OH was estimated from the double integration of the spectra.

### 2.6. Composition of Juices and Tea Infusion

The chemical composition of the juices and tea infusion was obtained from the literature data. The ginger (*Zingiber officinale* Rosc.) lyophilized water extracts contain pyrogallol, *p*-hydroxybenzoic, ferulic, *p*-coumaric, gallic and caffeic acids and vanillin as the main antioxidants [11]. A similar composition is expected in ginger juice, where strong Fe(III) chelating ligands are represented by pyrogallol, gallic and caffeic acids, which have a catechol moiety in their structure.

Blueberry juice is mainly composed of anthocyanins and anthocyanidins [12]; delphinidin, petunidin, cyanidin and their sugar containing derivatives have at least two adjacent phenolic OH groups that make them strong Fe(III) chelating ligands. Blueberry juice also contains chlorogenic acid [12], an ester of caffeic acid with quinic acid. This molecule contains two potential iron-coordinating residues: the catechol moiety of caffeic acid and an α-hydroxyacid moiety that stems from quinic acid.

The green tea infusion contains phenolics and flavonoids, with EGCG being the most abundant constituent [13].

Based on the compositions of ginger, blueberry juices and green tea infusion, two representative chemical compounds were selected: EGCG, which is the most abundant antioxidant of green tea, and GA, which is found in ginger juices but, containing a catechol moiety, also represents an important class of Fe(III) chelating ligands [14].

### 2.7. Statistical Analysis

The EC_50_ values and the corresponding 95% confidence intervals (CI 95%) were obtained with GraphPad Prism 8 using a straight-line modified model applied to a graph % inhibition vs. log (conc.). The lack of superimposition of the CI 95% of the EC_50_ values has been considered as a reliable criterion to distinguish statistically different values (*p* < 0.05) [15]. A one-way ANOVA was carried out to compare the DMPO–OH adduct intensities obtained with the four hydroxyl radical-generating systems without the addition of any extract or antioxidant compound. The mean separation was calculated by Tukey’s test at *p* ≤ 0.05.

## 3. Results and Discussion

### 3.1. Efficacy of the Different Hydroxyl Radical-Generating Systems: Intensity of the Blanks

**Figure 1:**
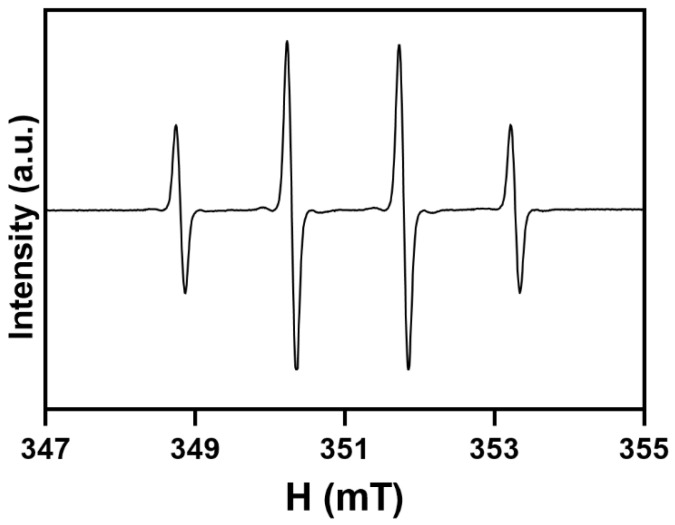
A typical EPR spectrum of the DMPO–OH adduct.

Regardless of the system used to produce the ^•^OH radicals, the DMPO–OH adduct was the only one detected. Figure 1 reports a representative spectrum, which is a four-line signal with hyperfine coupling constants *a*_N_ = *a*_H_ = 1.49 mT.

**Figure 2:**
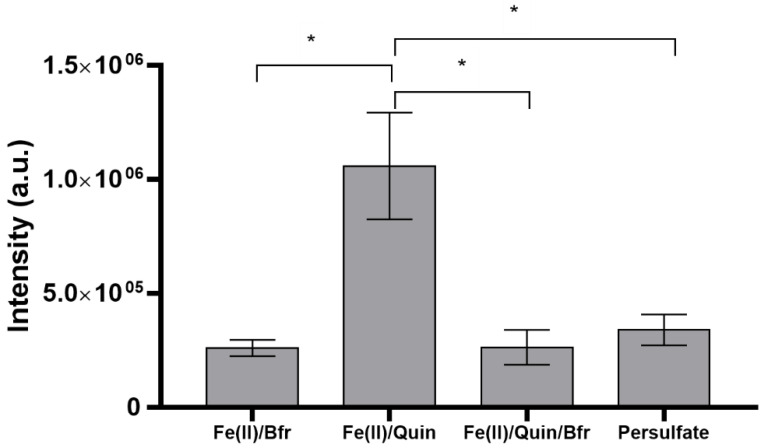
Number of hydroxyl radicals, measured as DMPO–OH signal intensity (a.u. arbitrary units) and produced with the four radical-generating systems. Fe(II)/Bfr: Fe(II)-phosphate buffer; Fe(II)/Quin: Fe(II)-quinolinic acid; Fe(II)/Quin/Bfr: Fe(II)-quinolinic acid-phosphate buffer; Persulfate: thermal degradation of peroxydisulfate. Bars marked by asterisks differ significantly by Tukey’s test (*p ≤* 0.05 *).

The presence of phosphate buffer and Quin influences the production and the entrapment of the hydroxyl radicals, as observed in our previous work [7]. A comparison of the adduct intensities of the blanks (the reference samples without the addition of the extracts) can be seen in Figure 2. The intensity of the blank in the Fe(II)/Quin system is significantly higher (ca. four times larger) than those measured in all the other systems, where the intensities are very similar. As previously observed in ref. [7], in the Fe(II)/Quin system, the production process of the hydroxyl radical is more effective than those in the other systems, because Fe(II) is stabilized by the complexation with quinolinic acid against the oxidation to Fe(III) by atmospheric O_2_. There are multiple effects of the phosphate buffer: it increases the pH of the solution, favoring the spontaneous oxidation of Fe(II), which becomes faster with increasing pH values [16], and by coordinating Fe(II)/Fe(III), it interferes with the radical-generating system. The final result is a decrease in the intensity of the DMPO–OH adduct, as previously reported [7]. Based on this consideration, it is expected that the radical scavenging capability of the antioxidants is lower in the Fe(II)/Quin system, because the production of hydroxyl radicals is more effective in comparison with the other systems. As a matter of fact, the ratio between the antioxidants and hydroxyl radicals is lower in this system in comparison with the others, and a higher EC_50_ value is therefore expected.

### 3.2. Hydroxyl Radical Scavenging Activity of Chemical Compounds and Plant Extracts

**Figure 3:**
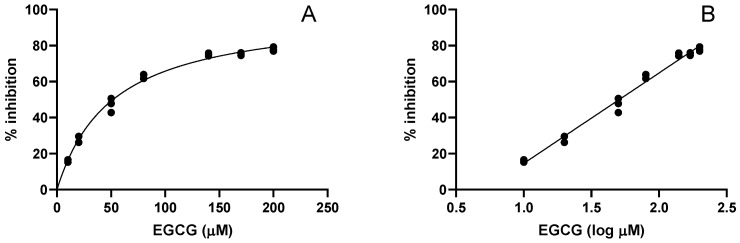
Percentage of inhibition as a function of EGCG concentration (**A**) and log EGCG concentration (**B**) in the Fe(II)/phosphate buffer system.

A graphic of the percentage of inhibition, calculated as reported in the experimental section, as a function of the EGCG concentration is reported in Figure 3A; this figure shows that there is no linear correlation between hydroxyl radical inhibition and EGCG concentration, but a flattening of the curve is observed at high concentrations. To make this relationship linear, the concentrations were converted into their corresponding log values (Figure 3B). The same behavior is observed in all the examined systems, so the latter type of graph was used for the determination of the EC_50_ values. Other experimental approaches could have been used for the determination of the EC_50_ values (i.e., other fitting procedures), but the purpose of this work was not the exact determination of the EC_50_ values. In fact, the goal was the determination of the EC_50_ values obtained in the four different hydroxyl radical-generating systems (three Fenton type reactions plus the thermal degradation of peroxydisulfate) and the comparison of these values to verify if there is an influence of the infusion/compound/juice (ICJ) composition.

**Figure 4:**
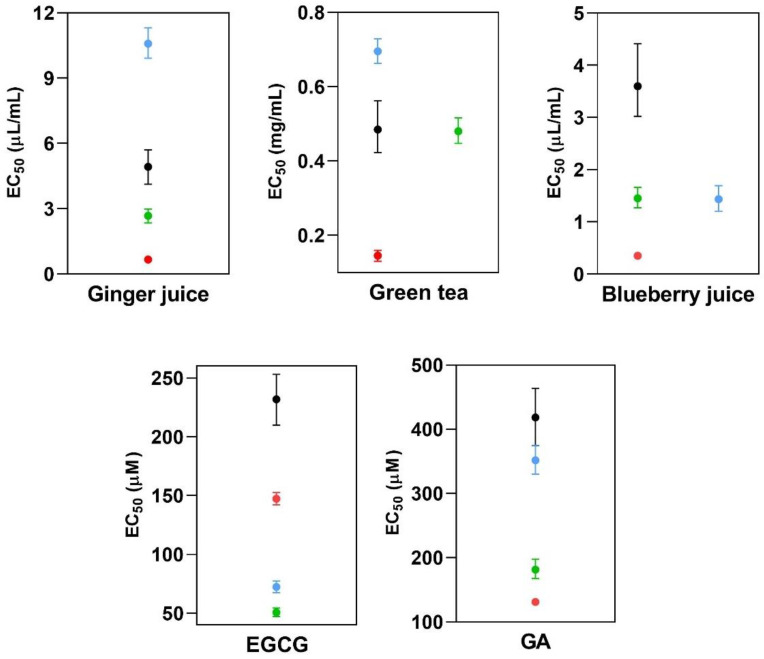
Graphical representation of EC_50_ and the corresponding CI 95% values for each plant juice, infusion and chemical compound. Each point represents a hydroxyl radical-generating system: Fe(II)/phosphate buffer (●), Fe(II)/Quin/phosphate buffer (●), Fe(II)/Quin (●), thermal degradation of peroxydisulfate (●).

The EC_50_ values and the corresponding 95% confidence intervals (CI 95%) are summarized in Table 1 and in Figure 4. The results demonstrate that the same extract or antioxidant compound, when tested with different hydroxyl radical-generating systems, shows remarkably different antioxidant capacities. The higher the EC_50_ value, the lower the antioxidant capacity of the ICJs. In the case of the ginger juice, for example, the antioxidant activity was low (EC_50_ 10.58 µL/mL) when measured with the Fe(II)/Quin/phosphate system and high (EC_50_ 0.6601 µL/mL) when measured with the Fe(II)/Quin system.

Figure 4 shows that the CI 95% intervals are never superimposed, except in the cases of blueberry juice measured in the systems with Fe(II)/phosphate buffer and Fe(II)/Quin/phosphate buffer and of green tea infusion measured in the system with Fe(II)/phosphate buffer and peroxydisulfate. An almost negligible superimposition of the CI 95% intervals is also observed for GA in the systems with Fe(II)/Quin/phosphate buffer and peroxydisulfate. The lack of superimposition of the CI 95% can be considered as a reliable criterion to distinguish statistically different values (*p* ≤ 0.05) [15]; therefore, if the CI 95% intervals are not superimposed, the corresponding EC_50_ values can be considered statistically different.

The differences in the EC_50_ values observed for the same ICJ could be explained by the fact that the composition of the ICJ can influence both the generation and the entrapment of the hydroxyl radical. The strong coordinating ligands of Fe(III) make the hydroxyl radical generation process less effective because Fe(II) is more easily oxidized without reacting with hydrogen peroxide. In fact, the reduction potential of the couple Fe(II)/Fe(III) depends on the coordinated ligand. Those stabilizing Fe(III), such as deferoxamine, decrease the reduction potential and completely inhibit ^•^OH production [17]; conversely, ligands such as 2,2′-bipyridine and phenanthroline stabilize Fe(II), increasing the reduction potential and making it unreactive [18]. When strong coordinating ligands of Fe(III) are present, the antioxidant capacity is overestimated because less intense signals of the DMPO–OH adduct are detected. This effect can be increased when the phosphate buffer is also present, since the pH increasing favors the deprotonation of the ligands, which are not able to coordinate in the acidic pH range (e.g., catechol derivatives) [19,20,21].

The effect of the pH, as previously discussed, can be used to explain the differences observed in the systems Fe(II)/Quin and Fe(II)/Quin/phosphate buffer (red and blue balls in Figure 4). With ginger and blueberry juices and with green tea infusion, a higher radical scavenging activity was measured in the system Fe(II)/Quin in comparison with Fe(II)/Quin/phosphate buffer, while with EGCG, the opposite is true. The expected behavior is that shown by EGCG, where the phosphate buffer effect is to promote the deprotonation of EGCG, favoring the Fe(III) coordination and giving an overestimated antioxidant activity. In the remaining cases (ginger juice and green tea infusion), a higher EC_50_ value is obtained with Fe(II)/Quin/phosphate buffer, while, when phosphate buffer is not present, the value is lower.

The behavior of these three plant matrices (blueberry and ginger juices, green tea infusion) can be rationalized considering that the hydroxyl radical generation is more effective in the Fe(II)/Quin system, while the addition of phosphate buffer decreases this efficacy (see the previous discussion about the intensity of blanks). A smaller number of hydroxyl radicals is generated in the system with phosphate buffer, and, therefore, the scavenging activity of the antioxidants is, in this case, somewhat overestimated.

The effect of Quin can be observed by comparing the results obtained in the systems Fe(II)/phosphate buffer and Fe(II)/Quin/phosphate buffer (green and blue balls in Figure 4). The EC_50_ values are significantly different in all cases, except with blueberry, for which they are practically coincident. When the two values are different, the EC_50_ obtained with the system Fe(II)/phosphate buffer is lower with EGCG but not with GA. If, in the matrices, no ligands capable of stabilizing Fe(II) are present, when phosphate buffer is present, this is more easily oxidized to Fe(III), which is eventually stabilized by complexation. The consequence is that a lower EC_50_ value would be measured when phosphate buffer is present, since, when variable amounts of the matrices are added, the intensity of the DMPO-OH adduct is decreased because of the radical scavenging activity of the antioxidants but also because of the decreased amount of Fe(II) capable of giving the Fenton reaction when ligands stabilizing Fe(III) are present.

GA is more antioxidant in the system Fe(II)/Quin/phosphate buffer in comparison with Fe(II)/phosphate buffer, that is, the presence of Quin makes GA more antioxidant. GA is known for being a strong chelating agent for Fe(III), giving catechol-like coordination, especially at neutral pH values [14]. Considering this, the opposite behavior should be expected.

With EGCG, the comparison of the systems is as expected, since the highest antioxidant activity is shown in the system Fe(II)/phosphate buffer due to the destabilization of Fe(II) when EGCG is present. When Quin is added, the destabilizing effect of Fe(II) vanishes, and a slightly lower antioxidant activity can be measured. When the phosphate buffer (maintaining Quin) is removed, the effect of the pH is diminished, the Fe(II) becomes more stable and the radical scavenging effect is lower. Finally, when the hydroxyl radical is generated by the thermal degradation of peroxydisulfate, the highest EC_50_ is obtained. In this case, the interference of the potential iron-coordinating ligands is lacking, and, therefore, this latter value can be considered as exempt from interferences with the radical-generating system.

The results of the antioxidant activity of GA and EGCG show that there is not a well-defined order for the EC_50_ values determined in the four hydroxyl radical generating systems. In the same way, considering the chemical complexity of plant matrices, we cannot expect to find the same well-defined order for the EC_50_ values measured in juices and tea infusion. In fact, these matrices contain several antioxidant compounds but also some other compounds that can interact with Fe(II) and/or Fe(III), influencing the hydroxyl radical-generating system.

The differences observed in the three Fenton-generating systems can be explained by the fact that there can be an interaction between the pool of antioxidants present in the vegetable matrices and the Quin and/or phosphate buffer. Quin can compete with the other potential iron-coordinating ligands present in the vegetable matrices. Polyphenolic compounds, besides being good iron-coordinating ligands, are also strong antioxidants, while organic acids can coordinate iron but do not exhibit antioxidant properties. On the other side, the effect of the phosphate buffer is to increase the pH of the solutions, favoring the spontaneous oxidation of Fe(II) to Fe(III), deprotonating potential iron ligands and favoring the metal coordination. These effects and their extents are different when phosphate buffer or Quin are present alone or together and also depend on the chemical composition of the vegetable matrices.

### 3.3. Pro-Oxidant Activity of EGCG

**Figure 5:**
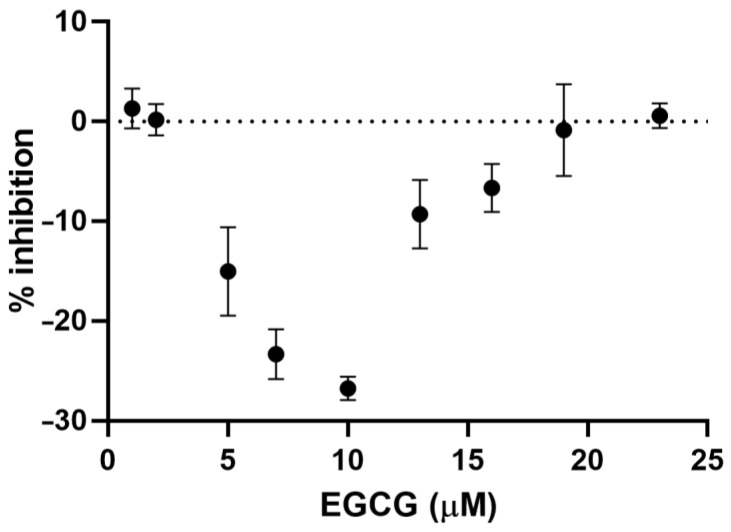
Percentage of inhibition as a function of the EGCG concentration measured in the system Fe(II)/Quin.

When examining the hydroxyl radical scavenging activity of EGCG in the Fe(II)/Quin system, we noticed that the % of inhibition at low EGCG concentrations was negative, that is, the intensity of the DMPO–OH adduct was higher than that of the blank. We decided to investigate this aspect in more detail and therefore used a lower concentration of EGCG to repeat these experiments. The results are shown in Figure 5.

At low concentrations, EGCG shows a pro-oxidant effect. The % of inhibition becomes negative if EGCG is added to the system up to ca. 10 µM and then increases again to reach zero, the same value of the blank (the reference sample without the EGCG added), at concentrations of 20–23 µM. Only at higher concentrations does the EGCG exhibit antioxidant effects. In the literature, the pro-oxidant effect of EGCG has already been described [22], but to the best of our knowledge, this is the first time that it has been described in relation to the Fenton reaction. The same behavior should be given by EGCG in the other Fenton systems and by all the other matrices examined in this work. In all the other cases, the concentrations used were not low enough and/or the pro-oxidant effect was not strong enough to be detectable. It is possible to exclude the possibility that hydroxyl radicals are generated by the direct reaction of EGCG with hydrogen peroxide, since, in this simple system, no DMPO–OH adduct was detected. Therefore, we should hypothesize that the larger production of hydroxyl radicals is likely due to a partial reduction of Fe(III) to Fe(II), assisted by Quin, which regenerates the reduced form which can then give the Fenton reaction.

## 4. Conclusions

As can be inferred from the above discussed results, a general trend of the EC_50_ values as a function of the hydroxyl radical-generating system cannot be observed for the juices/infusion (ginger, blueberry and green tea) or for the chemical compounds (GA and EGCG). Therefore, the first conclusion is that the results do not depend on the complexity of the matrices, that is, if they contain one or several compounds that exert the radical scavenging activity.

However, from a deeper analysis, it is clear that these assays do not measure the exact same effects. In fact, only the peroxydisulfate assay exactly measures the “radical scavenging activity against the hydroxyl radical” of the considered antioxidant (juice, infusion or chemical compound). In fact, as shown by Stasko et al. [5], the sulfate radical adduct is observed only in the very first stages of the reaction, while the reaction of SO_4_^−•^ with DMPO produces the same DMPO–OH adduct. In this assay, interferences of both the potentially iron-coordinating ligands and the pH effects are missing. However, this assay is not comparable with what takes place in the cellular system, where many bio-ligands are also present and could modify iron reactivity.

The Fenton reaction performed in the three different ways described above, coupled with the spin trapping of the hydroxyl radical by DMPO, measures the capacity of the considered matrix (juice, infusion, chemical compound) to oppose or to hinder the formation of the hydroxyl radical (by the reaction between Fe^2+^ and H_2_O_2_) and prevent its damages to cellular systems. Therefore, there are different steps by which the inhibition process can take place: (i) antioxidants that are also iron-binding ligands can change the redox potential of the couple Fe(II)/Fe(III), favoring or disfavoring the Fenton reaction; (ii) the pH increase to physiological values and the molecular oxygen dissolved in the solution favor the oxidation of Fe(II), unless the latter is stabilized by complexation, decreasing the ^•^OH yield of the Fenton reaction; (iii) hydrogen peroxide, being an oxidant, can react directly with antioxidants, causing their partial depletion; (iv) strong reducing agents can re-generate Fe(II) from Fe(III), thus increasing the number of hydroxyl radicals. These effects can be more or less noticeable depending on the amount of juice/infusion/chemical compound added to the reaction mixture.

Most of the antioxidants stabilize ferric iron and therefore exert a protective effect by disfavoring the Fenton reaction, which implies the presence of ferrous iron. Buffer employment, on one hand, better mimics the physiological pH of cellular systems and, on the other hand, makes the Fenton reaction less effective, favoring the spontaneous oxidation of iron. On the contrary, cellular systems are reducing environments; so, if free iron becomes available, it should remain in the reduced form, Fe(II), making the Fenton reaction more effective. The employment of Quin, which was proposed to simplify the experimental procedure, avoiding the degassing of the solution, can be useful but has no biological relevance.

Considering the results obtained in this work, it is clear that, in hydroxyl radical scavenging activity measurements, the chemical composition of the plant extracts and the hydroxyl radical-generating system affect the experimentally obtained EC_50_ values. In our opinion, the employment of phosphate buffer is strongly recommended in order to mimic the pH existing in physiological conditions. The use of quinolinic acid could be proposed to facilitate the experimental procedure since the spontaneous oxidation Fe(II) is prevented; moreover, it could be used to mimic the potential coordinating ligands present in cellular systems, which could interfere with the Fenton reaction.

In conclusion, the measurement of the hydroxyl radical scavenging activity with the two hydroxyl radical-generating systems, Fe(II)-phosphate buffer and Fe(II)-Quin-phosphate buffer, is advisable. The comparisons of the EC_50_ values of different plant extracts could be made only if these are obtained in the exact same experimental conditions.

Finally, it is advisable that the hydroxyl radical scavenging activity be measured in follow-up studies—for specific plant extracts—with cellular systems in vitro and that the results be compared with those obtained with the four hydroxyl radical-generating systems proposed in this work. In this way, it would be possible to establish the best protocol for these kinds of measurements.

## Figures and Tables

**Table 1:** EC_50_ and the corresponding CI 95% values obtained from the graphs % of inhibition vs. log (conc.) with nonlinear fit—straight line.

Extract	System	EC_50_	CI 95%	R^2^
Green tea infusion	Fe(II)/phosphate	0.4801 mg/mL	0.4476–0.5163	0.9624
	Fe(II)/Quin	0.145 mg/mL	0.1302–0.1591	0.9763
	Fe(II)/Quin/phosphate	0.6954 mg/mL	0.6625–0.7292	0.9809
	Peroxydisulfate	0.4846 mg/mL	0.4228–0.5625	0.9475
Blueberry juice	Fe(II)/phosphate	1.454 µL/mL	1.274–1.66	0.9312
	Fe(II)/Quin	0.3517 µL/mL	0.3008–0.4072	0.8926
	Fe(II)/Quin/phosphate	1.435 µL/mL	1.202–1.692	0.8565
	Peroxydisulfate	3.598 µL/mL	3.024–4.409	0.9270
Ginger juice	Fe(II)/phosphate	2.678 µL/mL	2.349–2.98	0.9427
	Fe(II)/Quin	0.6601 µL/mL	0.5341–0.7749	0.8919
	Fe(II)/Quin/phosphate	10.58 µL/mL	9.918–11.31	0.9778
	Peroxydisulfate	4.93 µL/mL	4.12–5.70	0.9186
GA	Fe(II)/phosphate	181.4 µM	167.4–197.8	0.9098
	Fe(II)/Quin	131.2 µM	126.2–136.3	0.9833
	Fe(II)/Quin/phosphate	351.8 µM	330.3–375	0.9670
	Peroxydisulfate	418.4 µM	374.7–463.4	0.9068
EGCG	Fe(II)/phosphate	50.78 µM	47.31–54.34	0.9835
	Fe(II)/Quin	147.3 µM	142.2–152.6	0.9795
	Fe(II)/Quin/phosphate	72.37 µM	67.67–77.47	0.9830
	Peroxydisulfate	231.9 µM	209.9–253.2	0.9808

## Data Availability

Not applicable.

## Sample Availability Statement

Samples of the compounds are not available from the authors.
